# The efficacy and safety of Chinese herbal medicine Shugan Sanjie decoction in the treatment of uterine fibroids: a systematic review and meta-analysis

**DOI:** 10.3389/fphar.2025.1508015

**Published:** 2025-04-17

**Authors:** Yang Zhang, Junfan Wei, Chanchan Gao, Shenyang Feng, Haiying Wang, Junjie Chai, Yini Zhu, Yuan Yuan, Lirong Ren

**Affiliations:** ^1^ The Seventh Clinical Medical College of Guangzhou University of Chinese Medicine, Shenzhen, China; ^2^ Department of Oncology, Zhongda Hospital Affiliated to Southeast University, Nanjing, China; ^3^ Department of Microbiology and Immunology, Medical School of Southeast University, Nanjing, China

**Keywords:** Chinese herbal medicine, shugan sanjie decoction, uterine fibroids, systematic review, meta-analysis

## Abstract

**Background:**

Shugan Sanjie Decoction (SGSJ) is a commonly used Chinese medicine prescription for the treatment of uterine fibroids (UFs). However, there is still a lack of evidence for its effects and safety. To systematically assess the efficacy and safety of SGSJ in conjunction with Mifepristone [MFP] or Leuprolide acetate [LA] for the treatment of UFs, thereby providing a reference for clinical medication.

**Objective:**

To systematically assess the efficacy and safety of SGSJ in combination with MFP or LA for the treatment of UFs, thereby providing a basis for clinical medication decisions.

**Methods:**

Eight digital medical databases were systematically searched to identify randomized controlled trials (RCTs) evaluating the use of SGSJ combined with MFP or LA for the treatment of UFs. The search spanned from the inception of each database to July 2024. Risk of Bias (ROB) 2.0 and RevMan 5.3 software were utilized for systematic review and meta-analysis. Eligible studies comprised RCTs comparing SGSJ plus MFP or LA with MFP or LA alone. The primary outcome was the Clinical Effective Rate (CER). Secondary outcomes included (1) Uterine Fibroid Volume (UFV) (2), Uterine Volume (UV) (3), Serum Sex Hormone Levels [Follicle-Stimulating Hormone (FSH), Luteinizing Hormone (LH), Estradiol (E2), Progesterone (P)], and (4) Traditional Chinese Medicine Syndrome Scores (TSS).

**Results:**

The meta-analysis comprised 12 RCTs with 952 participants. The results of meta-analysis showed that the total effective rate of SGSJ or combined with MFP or LA in the treatment of UFs [RR = 1.26, 95% CI (1.19, 1.34), *P* < 0.00001], which was statistically significant compared with the MFP or LA group and superior to the MFP or LA group (*P* < 0.05).

**Conclusion:**

At present, there are evidence shows that SGSJ combined with MFP or LA improves CER, reduces UFV, and modulates sex hormone levels. However, due to the poor methodological quality and high heterogeneity of the included trials, our conclusions should be interpreted with caution. Future studies should prioritize rigorous RCTs with standardized treatment protocols, extended follow-up, and comprehensive safety assessments to identify SGSJ as a reliable treatment option for UFs.

**Systematic Review registration:**

https://www.crd.york.ac.uk/PROSPERO/view/CRD42024506017

## 1 Introduction

Uterine fibroids (UFs), also known as uterine leiomyoma, are common benign monoclonal tumor affecting women of childbearing age (Stewart et al., 2017; De La Cruz and Buchanan, 2017; Ning et al., 2020), with risk factors including age, family history, hypertension (Mitro et al., 2024), obesity, diet, vaginal microbiota changes, environment, and so on (5–7). Patients with UFs always present different symptoms depending on the location and volume of the UFs. The closer the UFs are to the endometrium, the more severe the symptoms they cause, including excessive menstrual flow, prolonged menstrual periods, and in severe cases, may lead to a women being chronically anemic (Laughlin-Tommaso and Stewart, 2018; Lee and Stewart, 2023; Syed, 2022). In addition, UFs can lead to pelvic pain, frequent urination, urinary urgency, abdominal distension, abdominal pain and infertility in female patients (Lee and Stewart, 2023; Donnez and Dolmans, 2016).

The prevalence of UFs has been studied to be as high as 80%, but the actual prevalence may be higher considering the undiagnosed presence of asymptomatic patients (Laughlin-Tommaso and Stewart, 2018; Vannuccini et al., 2024). Current treatments for UFs are categorized as non-surgical and surgical (Marsh et al., 2024), with hysterectomy remaining the primary surgical treatment method, accounting for 75% of cases (Stewart et al., 2016). However, hysterectomies result in the loss of fertility in women of childbearing age and put women at increased risk for cardiovascular disease, cognitive impairment in later life, mental health and other diseases (Manyonda et al., 2020). And the high cost of surgery causes family economic burden and affects family harmony (Stewart et al., 2021; Walker and Stewart, 2005; Tulandi, 2007). Therefore, pharmacologic therapy among nonsurgical treatments has become the primary choice of many patients and physicians. Current pharmacologic measures include progesterone receptor modulators (PRMs), tranexamic acid, gonadotropin-releasing hormone (GnRH) agonists, and other symptom-relieving medications (mainly oral contraceptives, levonorgestrel, and intrauterine devices). Hormone therapy is usually the treatment of choice for most women, and studies have shown it to be effective in reducing UFs size and relieving clinical symptoms (Ali et al., 2023). There are certain adverse effects of drug therapy. For example, a large number of patients still suffer from adverse effects such as regeneration of UFs and endometrial hyperplasia, and patients need to increase drug dose due to long-term drug resistance (Fu et al., 2020; Stewart and Nowak, 2022). Therefore, researchers have sought safer alternative medications for the treatment of UFs. Conventional Western medical treatments have issues such as surgical risks and drug side effects, making Traditional Chinese Medicine (TCM), with its characteristics of multiple targets and low toxicity, a research hotspot (Zhang et al., 2010).

Shugan Sanjie Decoction (SGSJ) has the effect of soothing liver and clearing collaterals. Recently, the research on SGSJ for the treatment of UFs is increasing (Zhai, 2017). However, none of the articles have comprehensively evaluated the efficacy and safety of SGSJ for the treatment of UFs, which has resulted in limited evidence supporting SGSJ as an effective treatment option for UFs. Therefore, this study conducted a meta-analysis of randomized controlled trials (RCTs) of SGSJ for the treatment of UFs with the aim of systematically evaluating the efficacy and safety of SGSJ for the treatment of UFs, and providing a basis for the clinical treatment of UFs.

## 2 Materials and methods

**FIGURE 1:**
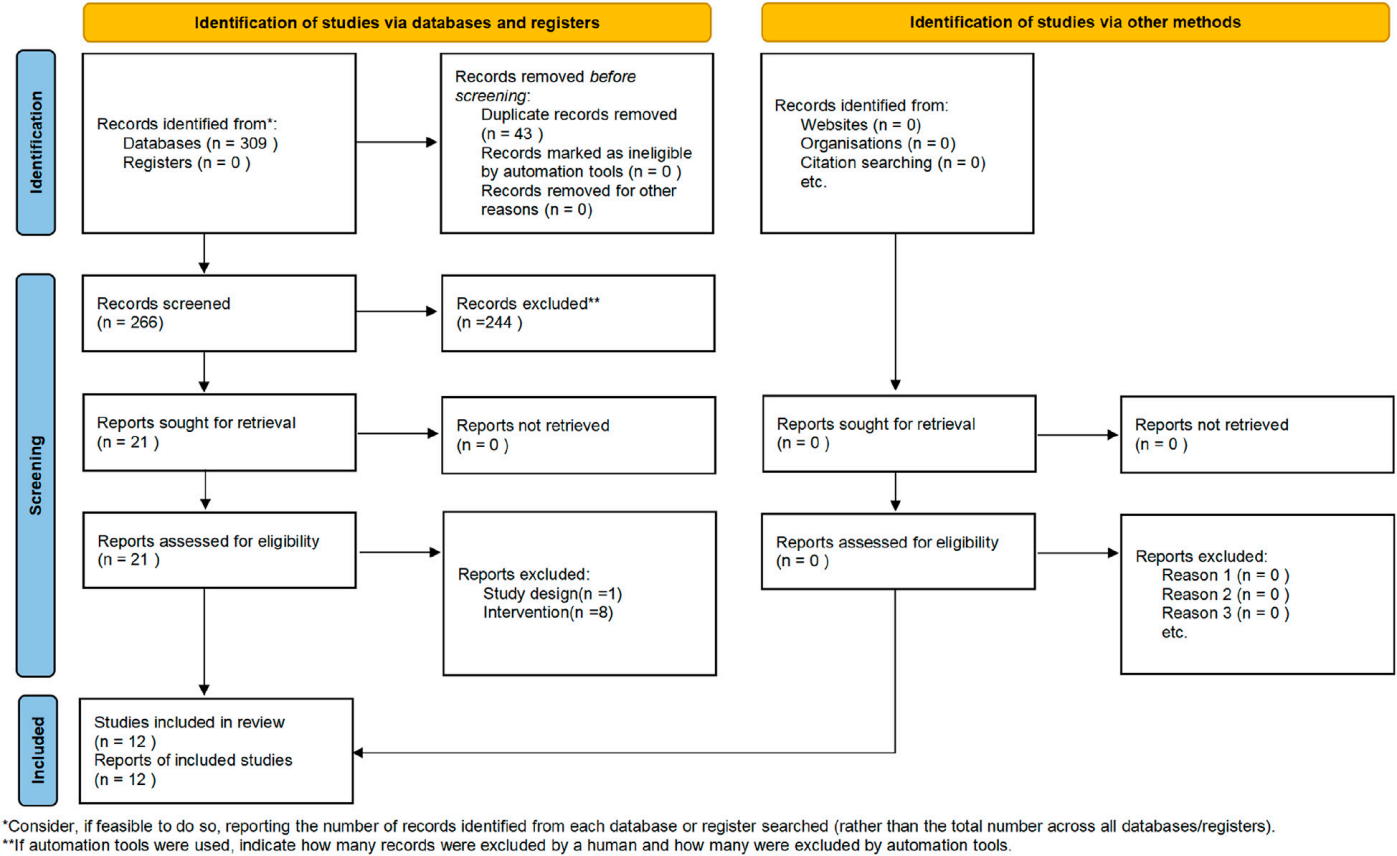
PRISMA flow chart of the selection process.

The present study adhered to the Preferred Reporting Items for Systematic Reviews and Meta-Analyses (PRISMA) guidelines (Page et al., 2021) (Figure 1).

### 2.1 Protocol registration

The present study followed the protocol registered in the International Prospective Register of Systematic Reviews (PROSPERO) (https://www.crd.york.ac.uk/prospero/), registration number: CRD42024506017. No personal data were collected for this study and ethical approval was not required.

### 2.2 Search strategy

We searched RCTs in the following eight databases from January 2024 to July 2024, including PubMed, Web of Science, Embase, Chinese Scientifc Journals Database (VIP), China National Knowledge Infrastructure (CNKI), Cochrane Library, ScienceDirect, Allied and Complementary MedicineDatabase (AMED), Wanfang, and Chinese Biological Medical Database (CBM). The following search terms were used in combination (Leiomyoma OR Fibroid Tumor OR Fibromyoma OR Fibroid OR Fibroid Uterus OR Fibroma, Uterine OR Fibroids, Uterine OR Leiomyoma, Uterine) AND (shugansanjie tang OR shugansanjie decoction) AND (Randomized Controlled Trials as Topic OR Clinical Trials, randomized OR Trials, Randomized Clinical OR Controlled Clinical Trials, Randomized).

### 2.3 Inclusion and exclusion criteria

#### 2.3.1 Types of studies

We included RCTs that have been published in both Chinese and English to evaluate the efficacy and safety of SGSJ in the treatment of UFs.

#### 2.3.2 Types of participants

Patients diagnosed with UFs, aged ≥18 years old.

#### 2.3.3 Types of interventions and comparators

This systematic review included studies on SGSJ and combination therapy (SGSJ and Mifepristone [MFP] or Leuprolide acetate [LA]). To ensure the reproducibility of the results, all herbal ingredients of SGSJ were described in accordance with the ConPhyMP (Consensus for Pharmacological Intervention of Medicinal Plants) guidelines. Botanical authentication, including species names, family, and identification methods, was documented where available. The following medication groups were compared in this study: a) the SGSJ and modified SGSJ groups with MFP or LA; b) the SGSJ group with placebo (same taste, shape, color, and odor as SGSJ) group; and c) combination therapy (SGSJ plus Western medicine) group with MFP or LA. In addition, SGSJ is composed of Bupleurum falcatum L [Apiaceae; Bupleuri radix], Cyperus rotundus L [Cyperaceae; Cyperi rhizoma], Salvia miltiorrhiza Bunge [Lamiaceae; Salviae miltiorrhizae radix et rhizoma], Paeonia lactiflora Pall [Paeoniaceae; Paeoniae radix rubra], Prunus persica (L.) [Rosaceae; Persicae semen], Corydalis yanhusuo [Papaveraceae; Corydalis rhizoma], Glycyrrhiza glabra L [Fabaceae; Glycyrrhizae radix et rhizoma], Commiphora myrrha (T.Nees) Engl [Burseraceae; Myrrha], Boswellia sacra Flück [Burseraceae; Olibanum], Conioselinum anthriscoides ‘Chuanxiong’ [Apiaceae; Chuanxiong rhizoma], Typha angustifolia L [Typhaceae; Typhae pollen], and Panax notoginseng (Burkill) F.H.Chen [Araliaceae; Notoginseng radix et rhizoma].

#### 2.3.4 Types of outcome measures

Clinical effective rate (CER) was the primary outcome. CER: The sum of the percentages of women with UFs that achieved cured, observably effective and effective. Cured: Clinical symptoms and signs disappeared completely, and B-ultrasound showed that UFs disappeared completely; Observably effective: The clinical symptoms improved significantly or basically disappeared, and the volume of myoma was reduced by ≥ 50%; Effective: Conscious symptoms improved, B-ultrasound showed myoma volume reduced by 25%–50%; Ineffective: Symptoms and signs did not improve, and B-ultrasound showed no reduction of fibroid volume or <25%. Secondary outcome measures (Stewart et al., 2017): Uterine fibroid volume (UFV) (De La Cruz and Buchanan, 2017). Uterine volume (UV) (Ning et al., 2020). Serum sex hormone levels [follicle-stimulating hormone (FSH), luteinizing hormone (LH), estradiol (E_2_), progesterone (P)] (Mitro et al., 2024). TCM syndrome scores (TSS).

#### 2.3.5 Exclusion criteria

The exclusion criteria for this study were as follows (Stewart et al., 2017): outcome indicators cannot be obtained (De La Cruz and Buchanan, 2017); repeated publications (Ning et al., 2020);incomplete data (Mitro et al., 2024); therapeutic measures combinated with other kinds of complementary and alternative treatments, such as acupuncture and moxibustion.

### 2.4 Study selection and data extraction

In this study, two independent evaluators, YZ and JW, searched the studies according to the search strategy and created a new database using the EndNote software to manage the studies. The title, keywords, abstract and the full text of the literature were screened by YZ and CG and the reasons for exclusion were recorded for further review. If two evaluators could not reach a consensus, the third evaluator (YZ or LR) would arbitrate. Data were collected by two reviewers (HW and JC), including the first author, publication date, number of included studies, sample size of trial group, treatment protocol details of trial group, treatment protocol of control group, follow-up time, post-treatment examination results, and adverse events were extracted. The extracted information was filled into the form we designed in advance, and the extracted data was cross-checked by two other reviewers (SF and YY) to ensure the accuracy of the data. If the information in the included article is unclear, it will be confirmed by the reviewer (LR) after inquiring by email.

### 2.5 Reporting quality assessment

To ensure the reproducibility and accurate interpretation of studies involving medicinal plant extracts, we employed the ConPhyMP (Heinrich and Jalil, 2023; Heinrich et al., 2022) checklist as a tool for assessing the quality of the 12 articles included in this study. Two researchers, SF and JC, independently extracted information from two checklists while being unaware of each other’s evaluations. A score of “1” or “0” was assigned based on whether the RCTs reported the relevant section/topic. A score of “0” indicated that the corresponding section/topic was not mentioned, whereas a score of “1” indicated that it was described by the author in their study (Heinrich and Jalil, 2023; Heinrich et al., 2022). Table 2, Table 3 presents both the number and percentage of ConPhyMP checklist items reported in these included studies.

**TABLE 1:** Summary characteristics of the included studies.

First author (year) [ref.]	Study design	Sample (T/C)	Age (T/C, years)	Interventions		Treatment duration	Outcomes	Funding
				Treatment	Comparator			
Haiyan Wu, 2021 (34)	RCT	30/30	47.61 ± 1.45/47.12 ± 2.72	SGSJ + MFP (10 mg/d, qd)	MFP (10 mg/d, qd)	12 weeks	CER	NR
Yanxia Chen, 2020 (35)	RCT	48/48	37.96 ± 4.63/38.47 ± 5.12	SGSJ + MFP(10 mg/d, qd)	MFP (10 mg/d, qd)	12 weeks	CER, UFV, UV, TSS	NR
Yanan Liu, 2020 (33)	RCT	41/40	36.32 ± 2.15/36.25 ± 2.06	SGSJ + MFP(10 mg/d, qd)	MFP (10 mg/d, qd)	2 weeks	CER, UFV	NR
Baozhen Wang, 2021 (41)	RCT	43/42	36.25 ± 2.06/41.56 ± 3.38	SGSJ + MFP(10 mg/d, qd)	MFP (10 mg/d, qd)	3 months	TSS, P	NR
Wei Ma, 2023 (36)	RCT	44/44	32.01 ± 5.42/31.85 ± 5.39	SGSJ + LA (3.75 mg/m, qw)	LA (3.75 mg/m, qw)	3 months	CER, UFV, UV, E_2_, LH, FSH	NR
Wenting Chen, 2022 (37)	RCT	37/37	43.99 ± 11.34/45.10± 15.34	SGSJ + MFP(10 mg/d, qd)	MFP (10 mg/d, qd)	3 months	CER, UFV, P	NR
Mingyuan Xu, 2022 (38)	RCT	16/16	33.27 ± 0.16/33.28 ± 0.31	SGSJ + MFP(10 mg/d, qd)	MFP (10 mg/d, qd)	3 months	CER, E_2_, LH, FSH, P	NR
Junyan Fan, 2021 (42)	RCT	60/60	40.62 ± 2.51/40.38 ± 2.64	SGSJ + MFP(10 mg/d, qd)	MFP (10 mg/d, qd)	12 weeks	CER, UFV, TSS	NR
Fumei Zhang, 2023 (39)	RCT	31/31	42.35 ± 3.52/42.32 ± 3.42	SGSJ + MFP(10 mg/d, qd)	MFP (10 mg/d, qd)	3 months	CER, UFV, P	NR
You Zhai, 2017 (25)	RCT	51/51	41.95 ± 6.02/42.58 ± 5.82	SGSJ + MFP(10 mg/d, qd)	MFP (10 mg/d, qd)	12 weeks	CER, UFV, P	NR
Bo Zhang, 2020 (43)	RCT	26/26	33 ± 0.8	SGSJ + MFP(10 mg/d, qd)	MFP (10 mg/d, qd)	12 weeks	UFV, TSS, P	NR
Li Zhou, 2019 (40)	RCT	50/50	41 ± 2.7/40 ± 2.5	SGSJ + MFP(10 mg/d, qd)	MFP (10 mg/d, qd)	8 weeks	CER, UFV, UV, E_2_, LH, FSH, TSS	NR

*Note*. Ref., reference; T, treatment grouping; C, comparator grouping; NR, not recorded.

**TABLE 2:** ConPhyMP checklist of information for reporting plant material and its initial processing.

Section/Topic	Item no.	Checklist item	n	ConPhyMP% (n/12)
Title and abstract	1	A clear and concise title including an informative abstract and balanced summary	11	91.7
Description of the botanical drug and taxonomic authentication	2	Botanical or morphological authentication of the plant material (desirable is a combination with DNA barcoding, e.g., PCR, RFLP, genome sequencing) and the information must be included in a separate section of Material and Methods, if applicable, combined with the information required under item 3	0	0
Description of the extract and extraction process	3	A separate section in Material and Methods, covers the relevant information on the material investigated, including the full species name(s), authorites and family; e.g., Salvia miltorrhiza Bunge [Lamiaceae; Salviae miltorrhizae radix et rhizoma], and on the processing and extraction of the crude drug including the traditional processing of the material used medicinally (fumigation, steaming, roasting, cooking, frying, *etc.*).	0	0
Documentation of the legal basis for collection and processing	4	Full compliance with the Nagoya protocol, CITES, and all associated treaties including phytosanitary regulations	12	100
Description of product characteristics, in case of a finished (commercial) product	5	Information on the characteristics of the commercial products including batch number and date of production/best by information and regulatory status.	0	0

**TABLE 3:** ConPhyMP checklist of items for conducting and reporting analytical methods relevant for extract type A.

Section/Topic	Item no.	Checklist item	n	ConPhyMP% (n/12)
Type of extract	1	A – Confirm that the species or botanical drug under investigation is covered in a monograph in one of the national or regional pharmacopoeias	12	100
Preferred/main methods for extract characterisation/chemical analysis	2a^*^	The description of the active ingredients in the botanical drug (if known) or analytical marker compounds as defined	12	100
	2b^*^	An analysis as defined in the monograph is needed if the extract has not been supplied with a certificate	0	0
	2c^*^	If the preparation was purchased, the manufacturer and certificate of analysis need to be included	0	0
	2a^#^	Triple chemical fingerprinting methods, each with one or more detection parameters	0	0
	2b^#^	Quantification of at least two marker compounds (unless this is not feasible, evidence needs to be provided), and justification of the choice of markers (if applicable)	0	0
Alternative methods for extract characterisation/chemical analysis	3a	Single chemical fingerprinting method with at least three different detection parameters (i.e., altered detection parameters, like TLC/HPTLC with different staining reagents and/or UV excitation wavelengths, HPLC-DAD/LCDAD with different wavelengths). The same applies to coupling MS or NMR to chromatographic techniques	0	0
	3b	Quantification of at least two marker compounds (unless this is not feasible, evidence needs to be provided), and justification of the choice of markers (if applicable)	0	0
Use of reference standards	4	(a) Direct overlay of the chromatogram of the sample with that of an officially specified reference standard (if applicable) (b) Chromatographic fingerprinting: Direct overlay of the chromatogram of the sample with that of official reference standards of the powdered plant material or the dry extract from the plant material	0	0
Comparison of different extracts/samples of the same plants	5	(a) Direct comparison of the chromatographic/spectroscopic system and/or scoring system for “similarity” to be followed	0	0

^*^Compliance with pharmacopoeial standards to be followed.

^#^Including either the preferred or alternative approaches for characterisation.

### 2.6 Risk of bias assessment

YZ and JW evaluated the quality of the included RCTs literature using the Cochrane risk bias assessment tool ROB 2.0 (Sterne et al., 2019), which included five types of bias involving randomization bias, bias from established interventions, bias from missing outcome data, bias from outcome measurements, and bias from selectively reported outcomes. Any disagreements are agreed upon after discussion with the other two reseachers (YZ and LR).

### 2.7 Data synthesis

Statistical analysis was carried out by RevMan 5.3 software (Wu et al., 2018), and the measurement indicators of the effects of the count data usually included Relative Risk (RR) and Odds Ratio (OR). Standardized Mean Difference (MD) and standardized Mean Difference (SMD) were used to describe effect sizes. In this study, RR or SMD were used. Both with 95% Confidence Interval (CI) to complete the analysis. The statistical heterogeneity among all studies was evaluated by *X* ^*2*^ and *I* ^*2*^ test with α = 0.05. When *P* > 0.1 and *I* ^*2*^ ≤ 50% of all studies, the included studies were considered to have no heterogeneity, and the Fixed effect model was adopted for analysis. When *P* ≤ 0.1 and *I* ^*2*^ > 50%, the included studies were considered heterogeneous, and random effect model was adopted for analysis (Huedo-Medina et al., 2006). If there is a large heterogeneity in the study, the source is initially explored through subgroup analysis, or further explored through sensitivity analysis. Sensitivity analysis was performed by sequentially removing each included study to assess the robustness of the meta-analysis results. Subgroup analyses were conducted based on disease duration (>12 months vs <12 months), treatment duration (>8 weeks vs. ≤8 weeks), medication combinations (SGSJ + MFP vs. SGSJ + LA) and doses of combined MFP (MFP≥20 mg/d vs. MFP <20 mg/d).

### 2.8 Quality assessment of the evidence

According to the outcome indicators, the quality of evidence in the included RCTS was evaluated using GRADE (Guyatt et al., 2008) (Grading of Recommendations, Assessment, Development and Evaluations) pro 3.6 software, which was divided into four levels: high, medium, low and very low, and the level of evidence was strictly evaluated.

## 3 Results

A total of 309 articles were searched in this study. Using literature management software, 43 duplicate articles were eliminated. By screening the titles, keywords, and abstracts, 244 articles with different study types, including animal experiments, experience, reviews, and research reviews, were eliminated. By screening the full text, 9 studies with inconsistent intervention types, study participants, study comparators and study outcome measures were deleted. A total of 12 articles were included in the final meta-analysis (Figure 1).

### 3.1 Study characteristics

12 RCTs involving 952 participants were included (Figure 1). All studies were conducted in China and published between 2017 and 2023. Among the included studies, one compared combination of SGSJ and LA with LA alone, while eleven compared combination of SGSJ and MFP with MFP alone. Regarding the dosage form and route of administration for SGSJ, all 12 studies utilized oral decoctions. For MFP and LA, eleven studies investigated oral tablets, and one study examined subcutaneous administration of LA. In a single study, the sample size ranged from 16 to 60 patients, and the treatment duration varied from 2 weeks to 3 months. Concerning the diagnostic criteria for UFs, four studies did not specify the basis, three adopted the 2017 Chinese Expert Consensus on the Diagnosis and Treatment of UFs, two referred to obstetrics and gynecology textbooks, one followed NCCN-2018 guidelines, and two used the 1994 Chinese Clinical Diagnosis and Treatment Criteria for UFs. Regarding the syndrome types of UFs, all 12 studies identified qi stagnation and blood stasis syndrome. See Table 1 for details.

### 3.2 Reporting quality

Table 2 provides an overview of the plant materials and their initial information checklist treatment of the included studies reveals, indicating that 38.3% of the items were reported. Upon reviewing the titles, it was found that only one study (Liu, 2020) lacked a clear and concise title (item 1). Additionally, none of the studies reported botanical or morphological identification of plant materials (item 2), relevant information on survey materials (including complete species name, authority, and family) (item 3), or information on the characteristics of commercial products (including batch number, production date, and regulatory status) (item 5). However, twelve studies were fully compliant with the Nagoya Protocol, CITES, and all relevant treaties, including the Plant Health Regulations (item 5).

Table 3 summarizes the analytical methods related to Class A extracts. In all the included studies, botanical drugs were documented in regional monographs or national pharmacopoeias (item 1). The active constituents of all the botanical drugs were identified (item 2a^*^). However, eight ConPhyMP items were not addressed in any of the literature (0%): the extract lacked a certificate (item 2b^*^), the manufacturer and assay certificate were not provided (Item 2c^*^), the use of triple fingerprinting was not mentioned (item 2a^#^), the quantification of at least two labeled compounds was not reported (item 2b^#^), single chemical fingerprinting with varying detection parameters was not described (Item 3a), the quantification of at least two labeled compounds was not specified (item 3b), reference standards were not utilized (item 4), and comparisons between different extracts or samples of the same substance were not conducted (item 5).

### 3.3 Risk of bias assessment

**FIGURE 2:**
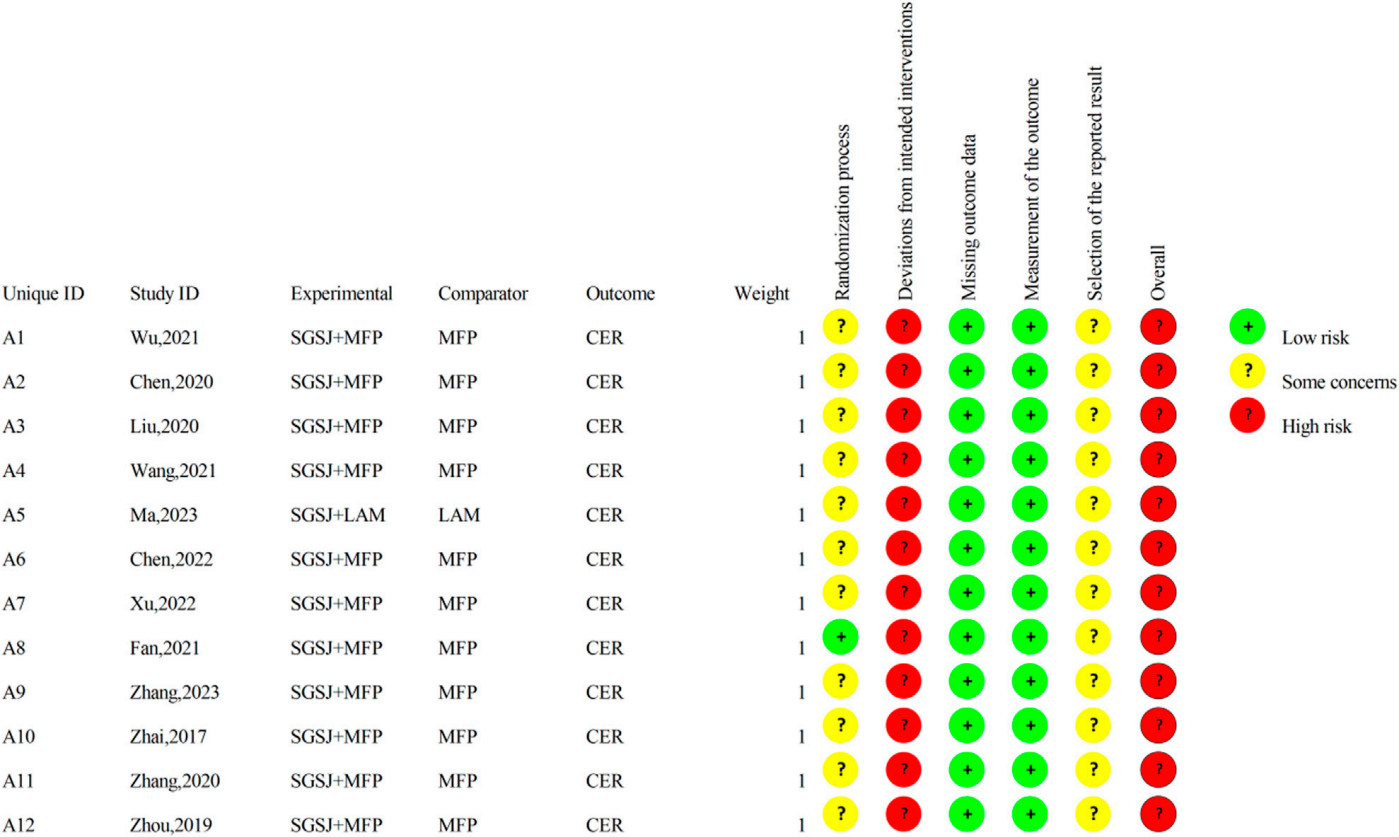
Risk-of-bias graph.

**FIGURE 3:**
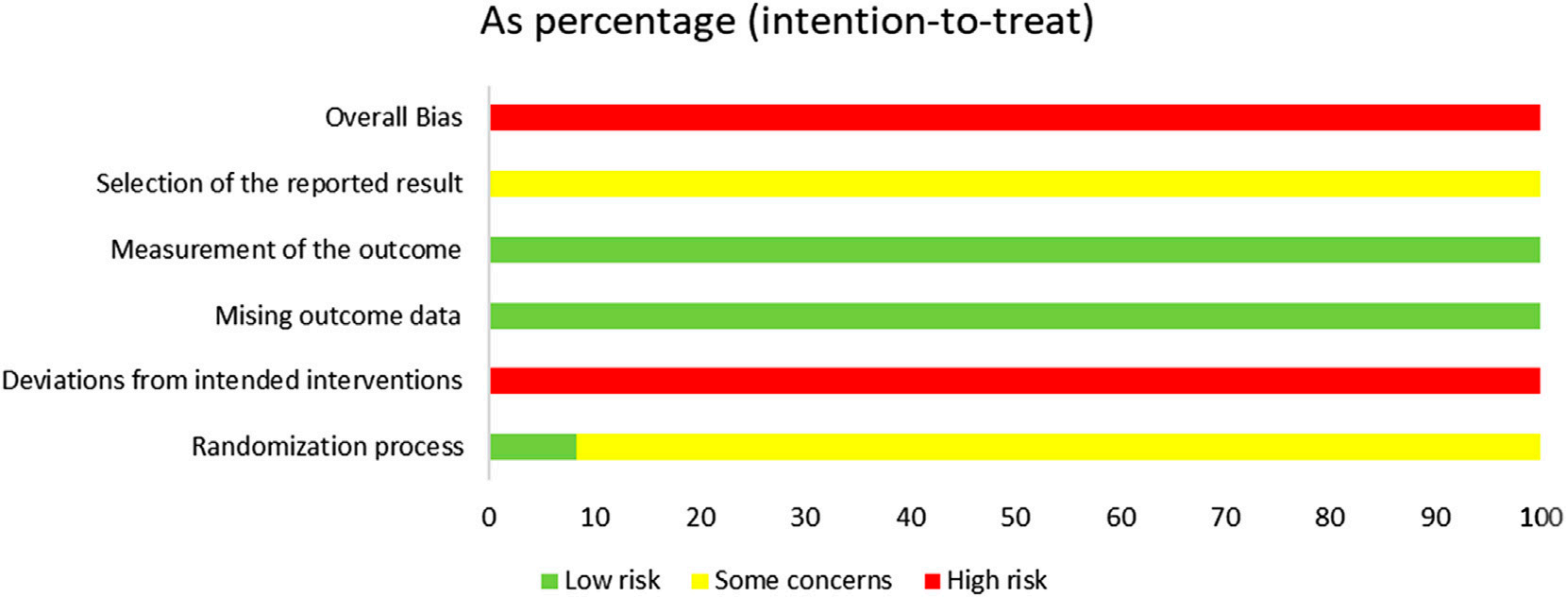
Risk-of-bias summary.

The risk of bias is illustrated in Figures 2, 3.

#### 3.3.1 Random sequence generation

Among the included studies, eight studies (Zhai, 2017; Wu, 2021; Chen, 2020; Ma, 2023; Chen, 2022; Xu, 2022; Zhang, 2023; Zhou, 2019) utilized the random number table method, two studies (Liu, 2020; Wang, 2021) employed the lottery randomization method, which were evaluated as having a low risk of bias. The remaining two studies (Fan, 2021; Zhang, 2020) did not specify the exact randomization method and were therefore assessed as having an unclear risk of bias.

#### 3.3.2 Allocation concealment

Among the 12 studies, only one study explicitly reported the concealment of allocation in the experiment, while the remaining 11 studies failed to provide such information. Consequently, one study (Fan, 2021) was rated as having a low risk of bias, whereas the risk of bias in the remaining 11 studies remained unclear.

#### 3.3.3 Blinding of participants and personnel

Of the 12 studies, only one (Fan, 2021) reported the implementation of double-blinding, however, it failed to provide a detailed description of the blinding method. Given that SGSJ was significantly different in appearance from MFP and LA, blinding participants and personnel to group allocation was not feasible. Consequently, all studies were evaluated as having a high risk of bias.

#### 3.3.4 Blinding of outcome assessment

Twelve studies failed to provide detailed descriptions regarding the blinding of outcome assessors; consequently, all twelve studies were rated as having an unclear risk of bias.

#### 3.3.5 Incomplete outcome data

All 12 studies reported complete outcome data and were evaluated as having a low risk of bias.

#### 3.3.6 Selective reporting

Due to the absence of explicit blinding, it was not feasible to ascertain whether the researchers had selectively reported the intervention after becoming aware of it. Consequently, all 12 studies were deemed to be at a high risk of bias.

#### 3.3.7 Overall bias

There was one instance of high risk of bias, two instances with unclear risks of bias, and two instances classified as low risks that were also cost-effective. Consequently, the overall assessment indicated a high risk of bias.

### 3.5 Meta-analysis results

#### 3.5.1 CER

**FIGURE 4:**
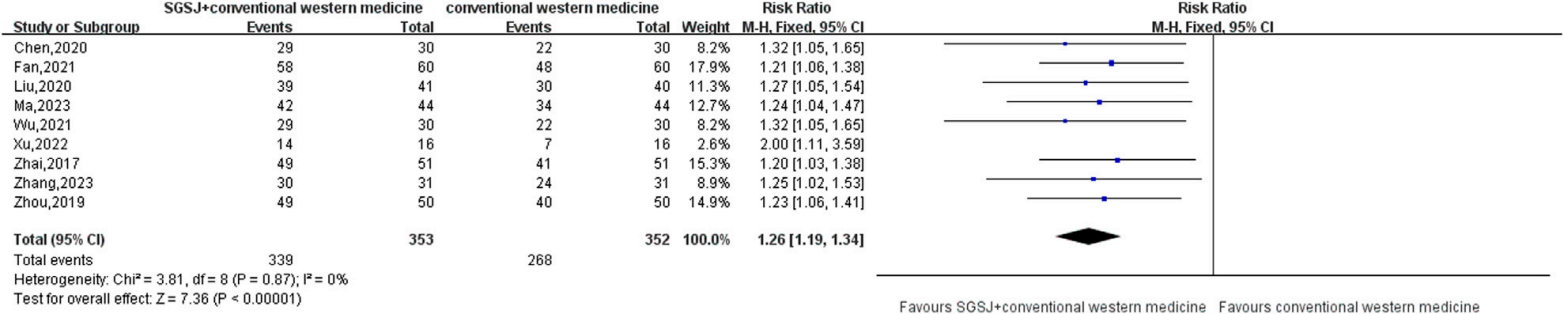
Forest plot of the clinical efficiency rate.

10 studies (Zhai, 2017; Liu, 2020; Wu, 2021; Chen, 2020; Ma, 2023; Chen, 2022; Xu, 2022; Zhang, 2023; Zhou, 2019; Fan, 2021) compared the total response rate of the test group and the control group, involving a total of 815 patients, most of which classified their CER as effective (effective, observably effective, cured) and ineffective. This data type is a binary variable, and RR combined effect size is selected. Heterogeneity test *P* = 0.87, *I* ^2^ = 0%, the conclusion is that there is no significant heterogeneity among the studies, so the fixed-effect model is selected for combined analysis. As shown in Figure 4, there was a statistically significant difference in the total effective rate between the SGSJ combined with MFP or LA group and the MFP or LA group [RR = 1.26, 95% CI (1.19, 1.34), *P* < 0.00001], which indicated that the SGSJ combined with MFP or LA group had a higher overall clinical effectiveness rate than the MFP or LA group in treating UFs. The results of sensitivity analysis by one-by-one elimination method showed no significant change, indicating that the study results were relatively stable.

#### 3.5.2 UFV

**FIGURE 5:**
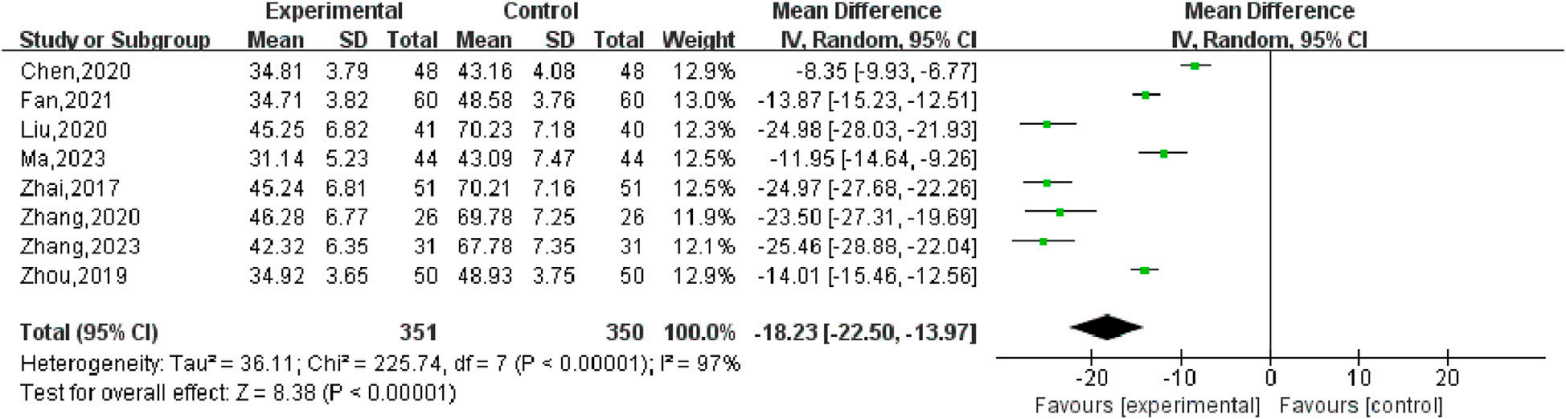
Forest plot of the UFs volume.

**FIGURE 6:**
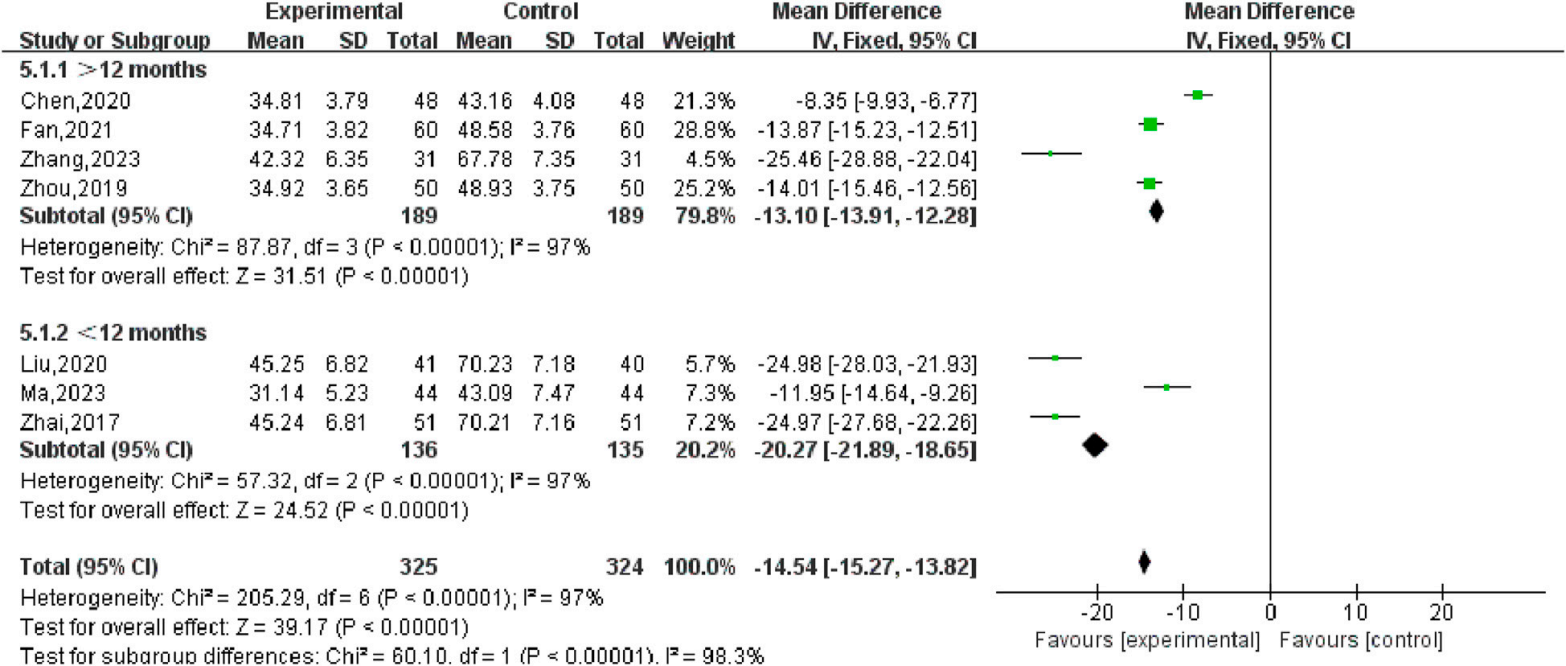
Forest plot of UFs volume disease course subgroup analysis.

**FIGURE 7:**
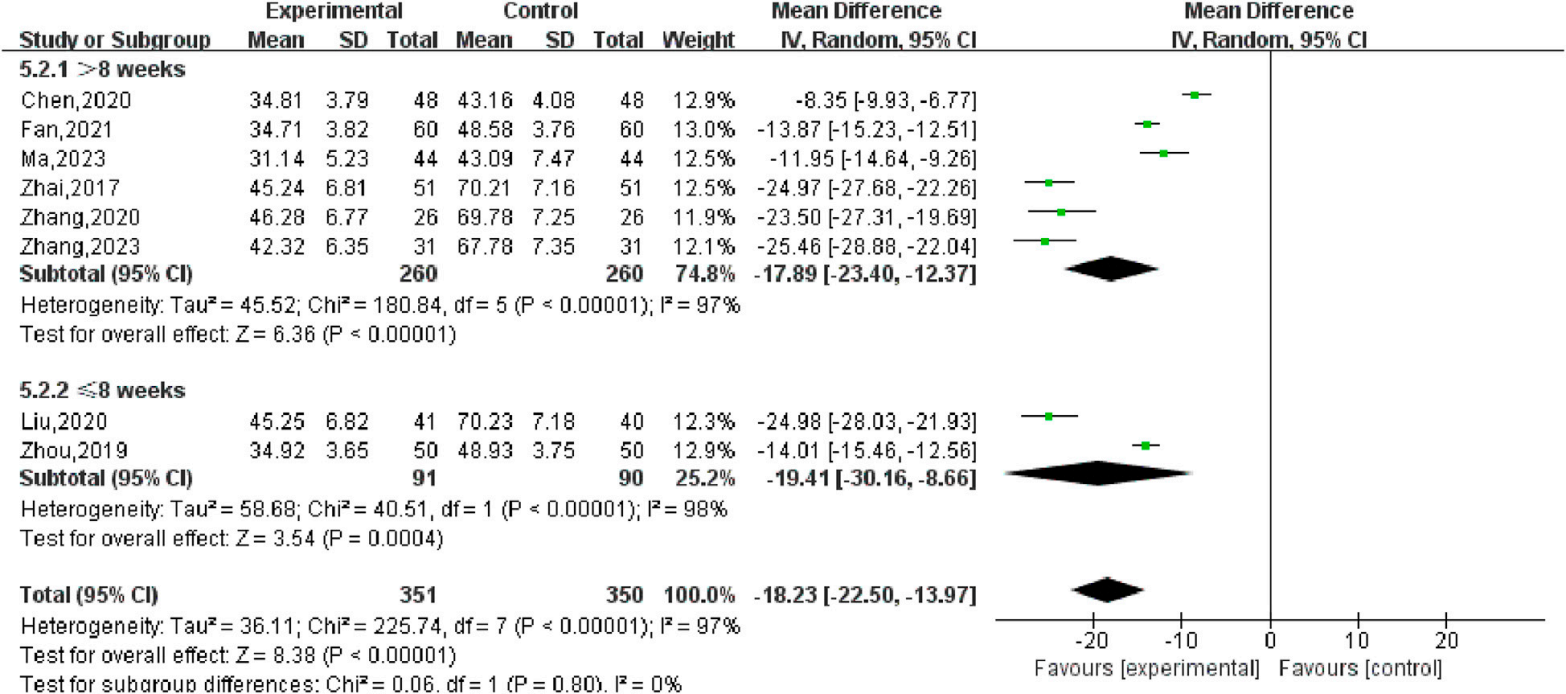
Forest plot of UFs volume treatment subgroup analysis.

Nine studies (Zhai, 2017; Liu, 2020; Chen, 2020; Ma, 2023; Chen, 2022; Zhang, 2023; Zhou, 2019; Fan, 2021; Zhang, 2020) reported changes in the volume of UFs before and after the treatment of SGSJ combined with MFP including 388 cases in the SGSJ combined with MFP group and 387 cases in the MFP group. Heterogeneity test *P* < 0.00001, *I* ^2^ = 97%, so the random effects model was adopted. As shown in Figure 5, there was a statistically significant difference in the volume of UFs between the SGSJ combined with MFP group and the SGSJ combined with MFP group [MD = −18.23, 95% CI (−22.50, −13.97), *P* < 0.00001], indicating that combined treatment could reduce the volume of UFs in patients more effectively. Sensitivity analysis was performed on these 9 literature, and no literature interfered with the results of this meta-analysis, indicating that this study has good stability. Subgroup analysis was conducted according to the duration of disease, the duration of disease was >12 months in 4 studies and <12 months in 3 studies. ① > 12 months: UFV in the SGSJ combined with MFP group was lower than that in the MFP group, the difference was statistically significant [MD = -13.10.95% CI (−13.91, −12.28), *P* < 0.00001]. ② < 12 months: UFV in the SGSJ combined with MFP group was lower than that in the MFP group, and the difference was statistically significant [MD = -20.27.95% CI (−21.89, −18.65), *P* < 0.00001], as shown in Figure 6. However, the pooled analysis showed significant statistical heterogeneity (chi-square = 205.29, degrees of freedom = 6; *I* ^2^ = 97%). Subgroup analysis was conducted according to the duration of treatment. ① > 8 weeks: UFV in SGSJ combined with MFP group was lower than that in MFP group, the difference was statistically significant [MD = -17.89.95% CI (−23.40, −12.37), *P* < 0.00001]; ②≤8 weeks: UFV in the SGSJ combined with MFP group was lower than that in the MFP group, and the difference was statistically significant [MD = −19.41.95% CI (−30.16, −8.66), *P* < 0.00001], as shown in Figure 7. However, the pooled analysis showed significant statistical heterogeneity (chi-square = 263.43, degrees of freedom = 8; *I* ^2^ = 97%). Significant heterogeneity (*I* ^*2*^ = 97%) persisted despite subgroup analyses based on disease duration and treatment duration, suggesting that other unconsidered factors (e.g., patient demographics, treatment adherence) may influence UFV outcomes.

#### 3.5.3 UV

**FIGURE 8:**
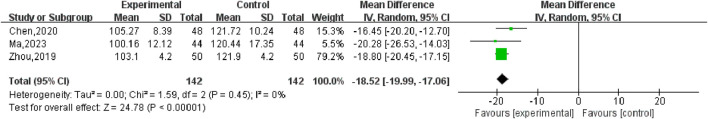
Forest plot of uterine volume.

Three studies (Chen, 2020; Ma, 2023; Zhou, 2019), with 284 participants, reported the effect of SGSJ in combination with MFP or LA compared with MFP or LA alone. Heterogeneity test *P* = 0.45, *I* ^2^ = 0%, fixed effect model was used for meta-analysis. The results showed that there was a statistically significant difference in UV between the SGSJ combined with MFP or LA group and the MFP or LA group [MD = −18.52, 95% CI (−19.99, −17.06), *P* < 0.00001] (Figure 8). The results of sensitivity analysis showed no significant change, indicating that the research results were relatively stable.

#### 3.5.4 TSS

**FIGURE 9:**
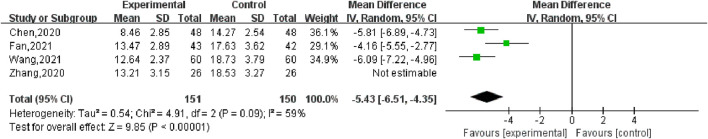
Forest plot of TCM syndrome scores.

Four studies (Chen, 2020; Wang, 2021; Fan, 2021; Zhang, 2020) reported the changes of Qi-stagnation and blood-stasis syndrome scores before and after treatment, including 177 cases in the SGSJ combined with MFP group and 176 cases in the MFP group. Heterogeneity test *P* = 0.09, *I* ^2^ = 59%, so the random effects model was used for meta-analysis. As shown in Figure 9, there was a significant statistically difference in symptom scores between the SGSJ combined with MFP group and the MFP group [MD = −5.43, 95% CI (−6.27, −4.60), *P* < 0.00001], which suggested that the SGSJ combined MFP group had a greater change in qi stagnation and blood stasis scores than the MFP group. Sensitivity analysis showed that when excluded (Fan, 2021), heterogeneity was reduced to 0%, but this did not overturn our conclusions, and the results were relatively stable.

#### 3.5.5 Follicle-stimulating hormone (FSH)

**FIGURE 10:**
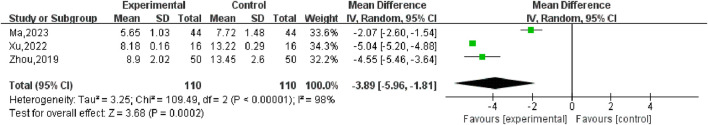
Forest plot of follicle-stimulating hormone.

**FIGURE 11:**
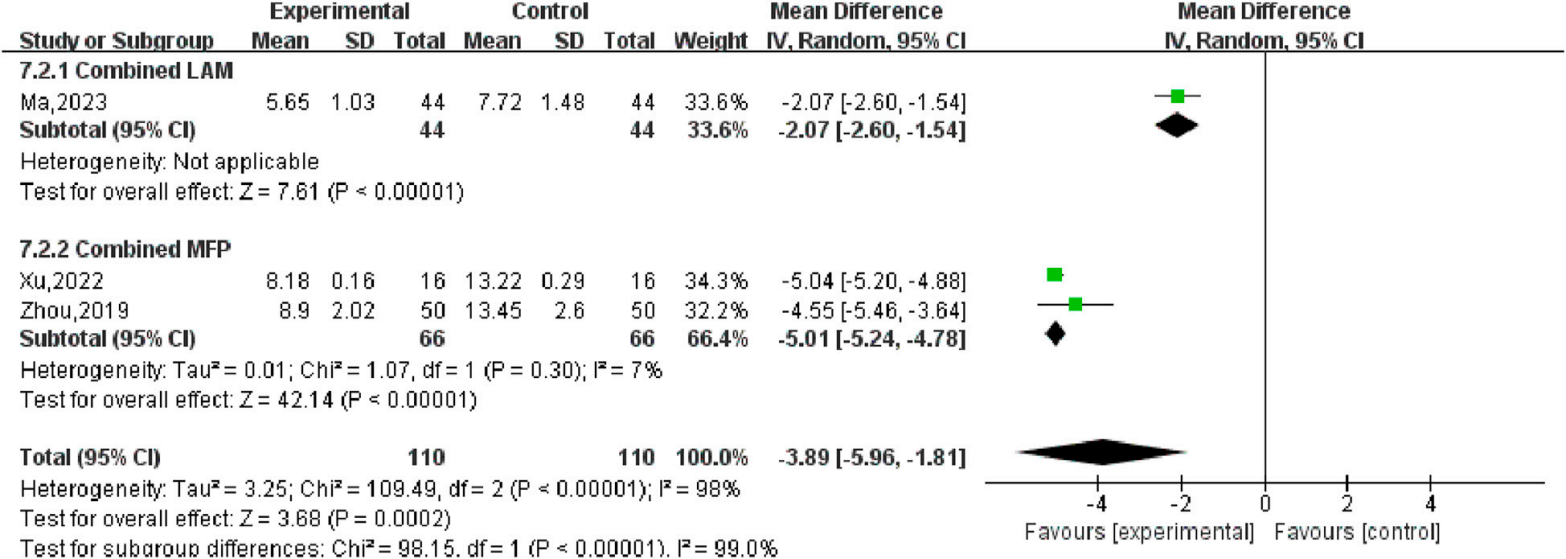
Forest plot of follicle-stimulating hormone combined drug use subgroup analysis.

Three studies (Ma, 2023; Xu, 2022; Zhou, 2019) reported changes in FSH values in patients before and after treatment, including 220 participants. Heterogeneity test *P* < 0.00001, *I* ^2^ = 98%, so the random effects model was used for meta-analysis, as shown in Figure 10, there was a statistically significant difference in symptom scores between the SGSJ combined with MFP or LA group and the MFP or LA group [MD = −3.89, 95% CI (−5.96, −1.81), *P* < 0.00001], which suggested that the SGSJ combined with MFP or LA group showed a greater change in FSH values than the MFP or LA group. Due to the large heterogeneity in the studies, we performed subgroup analysis based on the types of western medicines combined by SGSJ, with 1 study combining LA and 2 studies combining MFP. ① Combined with LA: the FSH of observation group was lower than that of control group, and the difference was statistically significant [MD = −2.07.95% CI (−2.06, −1.54), *P* < 0.00001]; ② Combined with MFP: the FSH in the observation group was lower than that in the control group, and the difference was statistically significant [MD = −3.89.95% CI (−5.96, −1.81), *P* < 0.00001], as shown in Figure 11. However, the pooled analysis showed significant statistical heterogeneity (chi-square = 109.49, degrees of freedom = 2; *I* ^2^ = 98%). FSH was more significantly reduced in the combined with MFP group compared to the combined with LA group, suggesting a possible drug-dependent interaction.

#### 3.5.6 Luteinizing hormone (LH)

**FIGURE 12:**
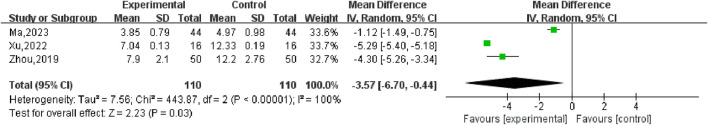
Forest plot of luteinizing hormone.

**FIGURE 13:**
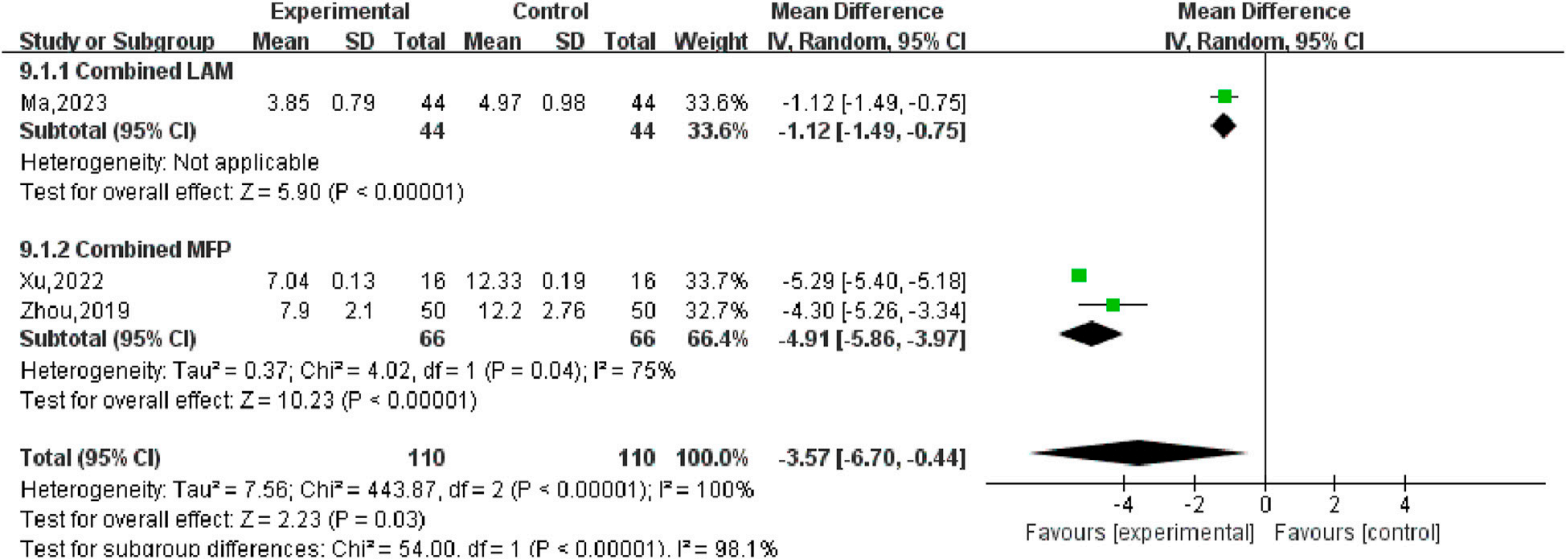
Forest plot of luteinizing hormone combined drug use subgroup analysis.

Three (Ma, 2023; Xu, 2022; Zhou, 2019) studies compared post-treatment LH levels in experimental and control groups, involving a total of 220 patients. Heterogeneity test *P* < 0.00001, *I* ^2^ = 100%, so the random effects model was used for meta-analysis, as shown in Figure 12, there was a statistically significant difference in symptom scores between the SGSJ combined with MFP or LA group and the MFP or LA group [MD = −3.57, 95% CI (−6.70, −0.44), *P* < 0.00001], which indicated that SGSJ combined with MFP or LA can significantly reduce the LH levels of patients. After sensitivity analysis, literature was removed one by one, and the final combined effect size did not change in direction, and the random effect model was used to merge. We performed a subgroup analysis based on based on the types of western medicines combined by SGSJ, with 1 study combining LA and 2 studies combining MFP. ① Combined with LA: LH in the observation group was lower than that in the control group, and the difference was statistically significant [MD = −1.12, 95% CI (−1.49, −0.75), *P* < 0.00001]; ② Combined with MFP: LH in the observation group was lower than that in the control group, and the difference was statistically significant [MD = −4.91.95% CI (−5.86, −3.97), *P* < 0.00001]. The results showed that TH was more significantly reduced in the combined with MFP group compared to the combined with LA group, suggesting a possible drug-dependent interaction (Figure 13).

#### 3.5.7 Progesterone (P)

**FIGURE 14:**
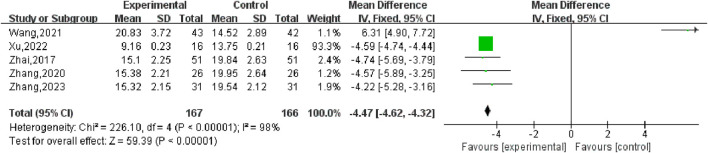
Forest plot of progesterone.

**FIGURE 15:**
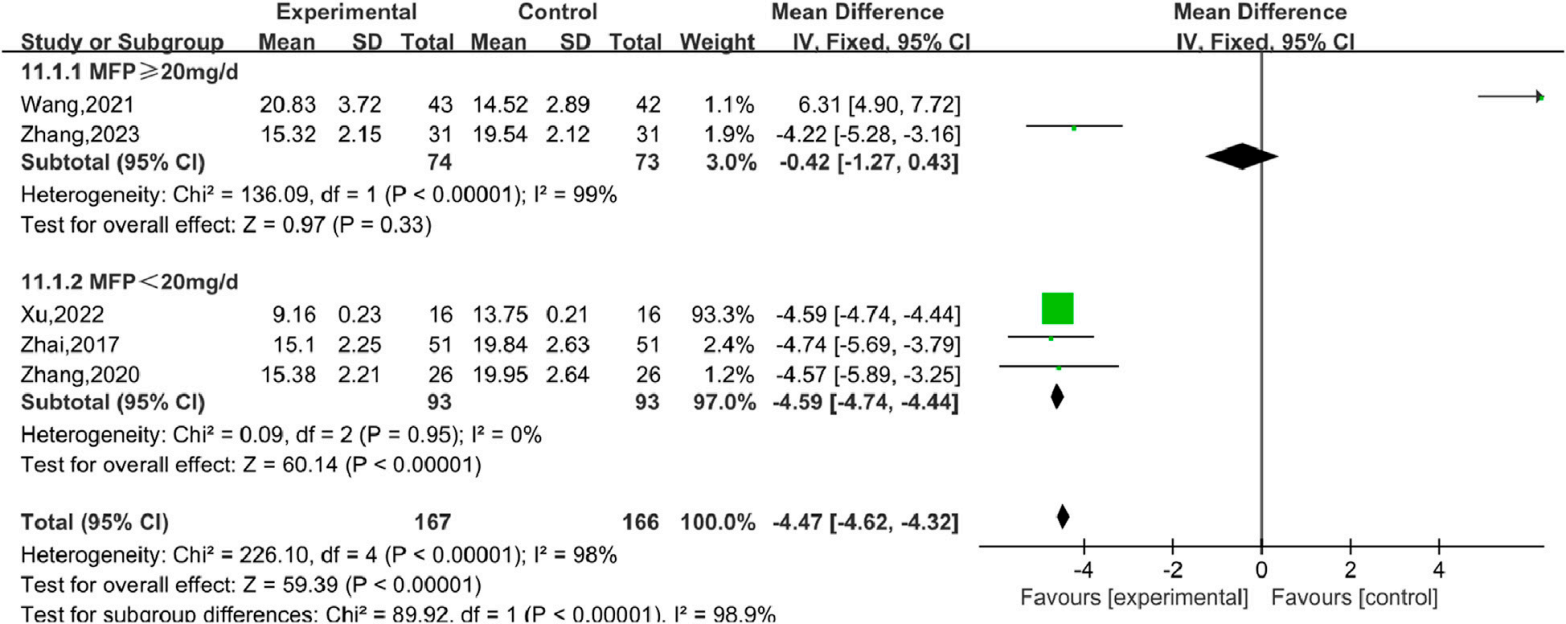
Forest plot of progesterone MFP dosage subgroup analysis.

Six studies (Wang, 2021), including 407 participants, reported the effect of combination therapy *versus* MFP alone in treating P. The heterogeneity of the pooled analysis was high (*P* < 0.00001, *I* ^2^ = 98%), so the random effects model was used in the meta-analysis. Comprehensive results show that the combined treatment can decrease the P [MD = 4.46, 95% CI (4.61, 4.32), *P* < 0.00001], (Figure 14). When Wang (2021) was excluded, the heterogeneity was reduced to 0%, but this did not overturn our conclusions, and the findings were relatively stable. We performed subgroup analyses based on different doses of combined MFP, with 2 studies having oral MFP≥20 mg/d and 4 studies having oral MFP <20 mg/d ① ≥20 mg/d: diamond shape across the inefficacy line, indicating that the difference between the experimental group and the control group was not statistically significant, that is, SGSJ combined with MFP or LA was no more effective in reducing P than the control group [MD = –0.42, 95% CI (−1.27, 0.43), *P* < 0.00,001]; ② < 20 mg/d: P in the observation group was lower than that in the control group, and the difference was statistically significant (MD = –4.59.95% CI (−4.73, −4.44), *P* < 0.00,001), as shown in Figure 15. Subgroup analysis showed that SGSJ combined with lower MFP doses (<20 mg/d) significantly reduced P levels, whereas no significant reduction was observed with higher MFP doses (≥20 mg/d).

#### 3.5.8 Estradiol (E_2_)

**FIGURE 16:**
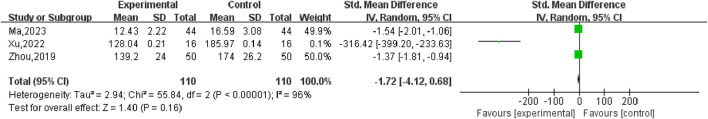
Forest plot of estradiol.

Three studies, including 220 participants, reported the effects of combination therapy *versus* conventional western drug therapy alone on E_2_. The heterogeneity of the pooled analysis was high (*P* < 0.0001, *I* ^2^ = 96%), so the random effects model was used in the meta-analysis. The comprehensive results showed that the difference between combined treatment and MFP or LA alone was not statistically significant E_2_ [Smd = −1.72.95% CI (−4.12, −0.68), *P* < 0.00001] (Figure 16). Sensitivity analysis showed that after removing [Xu, 2022] (Xu, 2022), the heterogeneity was reduced to 0%, which overruled our conclusion, indicating that the stability of the results of E_2_ meta-analysis was poor.

#### 3.5.9 Publication bias

In this meta-analysis, we tested publication bias by drawing funnel plots of eight outcome indicators, including total CER, UFV change, UV change, E_2_, P, LH and FSH levels, and TSS. In the funnel plots drawn by total clinical response rate, scatter points were more symmetrical, and there is little possibility of publication bias. The funnel plot of the other 7 outcome indicators showed asymmetric discrete distribution with publication bias.

#### 3.6 GRADE assessment

All studies included in this study were meta-analyzed and GRADE evidence assessment was evaluated. Problems of bias risk, limitation and inconsistency were found in the evaluation, due to the risk of bias in aspects such as low quality and accuracy of the literature, so that all the research studies were not eligible for GRADE upgrade and at least one was eligible for downgrade. Therefore, in the end, the highest GRADE evidence evaluation included in this study was only three of medium quality, and there were a lot of low-quality, very low-quality evidence. The quality of the evidence was reduced due to the risk of bias, inconsistency and imprecision. High heterogeneity between studies and unclear randomization methods resulted in low GRADE scores. It has long been a phenomenon that the quality evaluation of Chinese medicine research studies on methodology is generally not high. Due to the low quality of the studies, the trust of clinicians in research has decreased, which is one of the limitations of this study.

## 4 Discussion

UFs are common benign gynecologic tumors in women of fertility age (Donnez et al., 2012). Surgery and endocrine therapy may cause severe physical and psychological trauma, including impaired physiological function and body image problems (Dolmans and Donnez, 2024). Due to the untolerable side effects of conventional treatments, TCM has become a research hotspot for its multi-targeting and low-toxicity properties. However, differences in treatment methods and outcome assessment have limited the dissemination of existing TCM studies (Shi et al., 2023). In this study, evidence-based medicine and meta-analysis were used to evaluate the efficacy and safety of SGSJ for UF. However, the findings should be interpreted with caution due to methodological limitations and significant heterogeneity of the included studies.

The sensitivity analysis revealed that specific studies (e.g., Wang, 2021) had a disproportionate impact on heterogeneity. This may be due to differences in patient populations, intervention protocols, or outcome assessment methods.

### 4.1 Summary of evidence

To the best of our knowledge, this study represents the pioneering systematic review and meta-analysis evaluating the efficacy and safety of SGSJ combined with MFP or LA in the treatment of UFs in China. A total of 12 randomized controlled trials, encompassing 952 patients, were included in this analysis. The findings suggest that SGSJ in combination with MFP or LA significantly increases the CER and reduces the UFV compared with MFP or LA alone, demonstrating superior therapeutic efficacy. However, these findings should be interpreted with caution due to significant heterogeneity between studies and methodological quality limitations.

The results of meta-analysis showed that the overall CER of SGSJ combined with MFP or LA was higher than that of the control group. In addition, secondary outcome indicators, including UFV, UV, sex hormone levels (FSH, LH, P), and TSS, were significantly improved in the treatment group compared with the control group. However, there was no significant change in E_2_ levels.

Subgroup analyses showed that longer treatment duration (>8 weeks) resulted in greater UFV reduction and higher CER, as well as a significant reduction in heterogeneity, suggesting that sustained intervention may improve therapeutic efficacy. In addition, patients with UFs ≤12 months showed greater improvement than those with UFs ≤12 months, suggesting that SGSJ may exert a better therapeutic effect in early intervention. Subgroup analyses based on different drug combinations showed that SGSJ + MFP had more pronounced benefits in reducing UFV and modulating sex hormone levels compared with SGSJ + LA, and less heterogeneity was observed in the SGSJ + MFP subgroup, suggesting that the combination may be more consistently effective. In addition, MFP dose played a role in modulating the therapeutic effect, as high doses (≥20 mg/d) were associated with better regulation of P levels but also increased heterogeneity, whereas low doses (<20 mg/d) showed moderate efficacy with fewer reported adverse events, highlighting the need to optimize dosing strategies.

In addition, the high degree of heterogeneity observed in UFV (*I* ^*2*^ = 97%) and hormone-related outcomes (FSH, LH, P levels, *I* ^*2*^ > 90%) suggests that there is a great deal of variation between studies. Subgroup analyses based on treatment duration, disease duration, and drug combinations (SGSJ + MFP vs. SGSJ + LA) helped to minimize heterogeneity to some extent, but did not fully resolve it. This suggests that other factors, such as individual patient response variability and adherence differences, may influence the results. Future studies should standardize the dose and duration of treatment for MFP to minimize variability.

With regard to safety, limited adverse event data were available, with only one study reporting mild side effects such as rash and mild vaginal bleeding. In addition, the combination of SGSJ and MFP was associated with fewer adverse events than MFP alone, suggesting a potential protective effect of SGSJ against MFP-related side effects. However, the long-term safety of SGSJ remains uncertain due to limited reports of adverse reactions and lack of long-term follow-up data.

### 4.2 Reporting quality

Compliance with the ConPhyMP tool was suboptimal in RCTs of SGSJ combination therapy for UFs. The average proportion of ConPhyMP tool usage across all included studies was only 38.3% and 20%, respectively. Specific issues identified include (Stewart et al., 2017): inadequate descriptions of plant materials, lacking botanical or morphological identification, which hinders readers’ understanding of the morphological structure and functional relationships of botanical drugs (De La Cruz and Buchanan, 2017); omission of full species names, authorities, and family information, which impairs patients’ ability to accurately identify botanical drugs (Ning et al., 2020); lack of detailed information about commercial products, reducing transparency and affecting readers’ assessment of safety. Regarding the ConPhyMP for extract type A, the following deficiencies were noted (Stewart et al., 2017): absence of a certificate for the extract (De La Cruz and Buchanan, 2017); lack of manufacturer and analytical certificates (Ning et al., 2020); failure to perform triple chemical fingerprinting of botanical drugs (Mitro et al., 2024); quantification of at least two labeled compounds was not conducted (Yang et al., 2022); none of the reports utilized a single chemical fingerprinting method (Silberzweig et al., 2016); labeled compounds were not quantified, and no justification was provided for these omissions. The inconsistent reporting of SGSJ components across studies weakens the reliability of results. Future research should adhere to international standards (e.g., ConPhyMP) to provide consistent botanical authentication, extraction details, and quality control measures.

### 4.3 The impact of bias risk on outcomes

#### 4.3.1 Selection bias and performance bias

Only one study (Fan, 2021) reported the implementation of blinding procedures. The remaining studies did not provide detailed protocols regarding blinding, concealment of the randomization process, or sample size calculations. Non-transparent randomization methods and inadequate concealment of group assignments may have resulted in imbalanced baseline characteristics between groups. What’s more, the persistence of high heterogeneity in UFV outcomes, even after subgroup analysis, suggests potential confounding factors such as individual patient response variability and differing intervention adherence. Further research is needed to investigate additional factors that may influence treatment response, such as baseline fibroid characteristics, hormonal status, and genetic variations. Additionally, the absence of blinding for participants and research personnel could have led to differential intensities of adjunctive therapy or care provided to the intervention and control groups. For instance, patients in the placebo group might have exhibited reduced adherence due to awareness of their group allocation. Consequently, the combined effect size may be either overestimated or underestimated. In unblinded studies, the treatment effect in the intervention group may be exaggerated by a placebo effect or observer expectations. Future studies should ensure strict blinding of participants and assessors to minimize performance and detection bias. In addition, appropriate allocation concealment methods (e.g., sealed opaque envelopes or centralized randomization) should be used to improve methodological quality.

#### 4.3.2 Reporting bias and measurement bias

The efficacy evaluation indicators in all studies were insufficiently comprehensive, with critical outcomes such as menstrual volume, long-term efficacy, and recurrence rate remaining underreported. Standardized tools for assessing menstrual volume were not utilized. Consequently, the impact of the interventions on long-term outcomes remains unclear. Incomplete information for clinical decision-making: The absence of key outcomes may mislead clinical practice and decision-making.

#### 4.3.3 Follow-up bias and time effect bias

None of the studies provided long-term follow-up data exceeding 6 months. The high rate of loss to follow-up, coupled with the lack of intention-to-treat analyses, may have led to an underestimation of adverse events or recurrence rates. Short-term treatment effects might not accurately represent sustained efficacy or delayed side effects of the intervention. Consequently, this could result in an overestimation of short-term benefits (e.g., an intervention that is initially effective but subsequently fails) and inadequate safety assessments for identifying long-term adverse effects.

### 4.4 Implications for research

In view of the risk of bias mentioned, we proposes several recommendations for future research. Firstly, future RCTs should provide detailed descriptions of their randomization methods, encompassing simple, block, stratified, and cluster randomization techniques. Secondly, it is advised that experimental studies on botanical drugs adhere strictly to the ConPhyMP checklist in both design and reporting. Additionally, rigorous blinding procedures must be implemented to ensure blinding of participants, researchers, and observers. However, the preparation and evaluation of TCM placebos present greater challenges compared to chemical drugs due to the unique taste and diverse odors of TCM formulations. Consequently, future research should explore the development of safe and comparable TCM placebos. Furthermore, all adverse events in each study group should be meticulously documented in accordance with the CONSORT 2010 Statement (48), and the duration of clinical trials should be extended to assess the long-term safety of SGSJ in treating UFs. In addition, future studies should systematically evaluate the dose-response relationship between MFP and SGSJ to refine the optimal treatment protocols.

### 4.5 Strengths and limitations

Our study possesses several notable strengths. Firstly, despite the increasing number of clinical studies investigating the treatment of UFs, there has been no systematic review or meta-analysis focusing on the combination of SGSJ and MFP or LA for UFs. This study represents the first comprehensive evaluation of SGSJ, thereby addressing a significant gap in the evidence base for its use in treating UFs. Secondly, our interpretation of the results is conducted with a high degree of caution. We conducted subgroup analyses to identify sources of heterogeneity and performed sensitivity analyses and publication bias tests. Additionally, we incorporated both subjective (CER, TSS) and objective (UFV, UV, LH, FSH, E_2_, P, and adverse effects) outcome measures.

Although best efforts have been made to refine the methodology of this study, inevitably there are still several limitations of our study. First, Adverse drug effects are inadequately recorded, only one study (Ma, 2023) reported adverse effects, and none included monitoring of liver and kidney function, hormone levels, or long-term complications such as cancer risk. This significantly limits the reliability of any safety conclusions drawn. Second, the follow-up duration was insufficient: all studies had a follow-up period of less than 6 months, precluding an accurate assessment of both the persistence of efficacy and potential long-term toxicity. In addition, the study populations were highly homogeneous: all studies were conducted in China, which may restrict the generalizability of the findings to other ethnic groups or healthcare systems.

Finally, the most significant limitation of this study is the lack of efficacy in assessing drug safety. This is mainly attributed to the following reasons: firstly, only one study (Ma, 2023) reported adverse effects, while the remaining studies provided no data on adverse events. This absence of information may stem from selective reporting bias or inadequate monitoring protocols. Secondly, none of the studies included long-term follow-up periods exceeding 6 months. What’s more, the reporting of Chinese medicine components is inconsistent (such as lack of botanical identification and standardized extract information), affecting the credibility of the results. Consequently, several critical safety questions remain unaddressed (Stewart et al., 2017): Long-term organ toxicity: it remains unclear whether cumulative damage to liver and kidney function exists with SGSJ (De La Cruz and Buchanan, 2017). Effects on hormone homeostasis: it still unclear whether long-term use leads to abnormal fluctuations in FSH or LH levels (Ning et al., 2020). Reproductive and pregnancy risks: Insufficient data on exposure during pregnancy to assess potential effects on fetal development.

## 5 Conclusion

At present, there are evidence shows that SGSJ combined with MFP or LA improves CER, reduces UFV, and modulates sex hormone levels. However, due to the poor methodological quality and high heterogeneity of the included trials, our conclusions should be interpreted with caution. Future studies should prioritize rigorous RCTs with standardized treatment protocols, extended follow-up, and comprehensive safety assessments to identify SGSJ as a reliable treatment option for UFs.

## Data Availability

The original contributions presented in the study are included in the article/supplementary material, further inquiries can be directed to the corresponding authors.

## References

[B1] Ali M. Ciebiera M. Wlodarczyk M. Alkhrait S. Maajid E. Yang Q. (2023). Current and emerging treatment options for uterine fibroids. Drugs 83 (18), 1649–1675. 10.1007/s40265-023-01958-6 37922098

[B2] Chen L. Chen H. Yang Q. Jiang Y. Liu L. Yu H. (2022). Guizhi Fuling Capsule inhibits uterine fibroids growth by modulating Med12-mediated Wnt/β-Catenin signaling pathway. J. Ethnopharmacol. 290:115115. 10.1016/j.jep.2022.115115 35181487

[B3] Chen W. (2022). Clinical effect of Shugan Sanjie Decoction on the syndrome of qi stagnation and blood stasis in uterine fibroids. Inn. Mong. J. Traditional Chin. Med. 41 (03), 24–25. 10.16040/j.cnki.cn15-1101.2022.03.030

[B4] Chen Y. (2020). Effect of Shugan Sanjie Decoction on uterine volume and menstrual volume in patients with uterine fibroid syndrome of Qi stagnation and blood stasis. Chin. J. Ethnomedicine Ethnopharmacy 29 (20), 117–119.

[B5] De La Cruz M. S. Buchanan E. M. (2017). Uterine fibroids: Diagnosis and treatment. Am. Fam. Physician 95 (2), 100–107. Cited in: Pubmed; PMID 28084714. 28084714

[B6] Dolmans M. M. Donnez J. (2024). Solving the mysteries surrounding uterine fibroids: are we almost there? Fertil. Steril. 122(1):4–5. Declaration of Interests M.M.D. has nothing to disclose. J.D. has nothing to disclose. Epub 20240511. 10.1016/j.fertnstert.2024.05.144 38740322

[B7] Donnez J. Dolmans M. M. (2016). Uterine fibroid management: from the present to the future. Hum. Reprod. Update. 22(6):665–686. Epub 20160727. 10.1093/humupd/dmw023 27466209 PMC5853598

[B8] Donnez J. Tomaszewski J. Vázquez F. Bouchard P. Lemieszczuk B. Baró F. (2012). Ulipristal acetate versus leuprolide acetate for uterine fibroids. N. Engl. J. Med. 366(5):421–432. 10.1056/NEJMoa1103180 22296076

[B9] Fan J. (2021). Clinical study of Shugan Sanjie Decoction treating syndrome of Qi-stagnation and blood-stasis in uterine fibroids. Shenzhen J. Integr. Traditional Chin. West. Med. 31 (12), 74–76. 10.16458/j.cnki.1007-0893.2021.12.031

[B10] Fu Y. Fan Y. Fan W. Lv Y. Ai S. Yu C. (2020). Efficacy and safety of traditional Chinese herbal formula combined with western medicine for uterine fibroid: a protocol for systematic review and meta-analysis. Med. Baltim. 99, e22039(36). 10.1097/md.0000000000022039 PMC7478476 32899062

[B11] Guyatt G. H. Oxman A. D. Vist G. E. Kunz R. Falck-Ytter Y. Alonso-Coello P. (2008). GRADE: an emerging consensus on rating quality of evidence and strength of recommendations. Bmj. 336, 1170–1173. 924-6. 10.1136/bmj.39504.506319.80 18436948 PMC2335261

[B12] Harris H. R. Petrick J. L. Rosenberg L. (2022). The epidemiology of uterine fibroids: where do we go from here? Fertil. Steril. 117(4):841–842. Epub 20220309. 10.1016/j.fertnstert.2022.01.037 35277259

[B13] Heinrich M. Jalil B. (2023). From the CONSORT to the ConPhyMP statement and beyond-how to ascertain best practice. Front. Pharmacol. 14:1338710. 10.3389/fphar.2023.1338710 38149050 PMC10750347

[B14] Heinrich M. Jalil B. Abdel-Tawab M. Echeverria J. Kulić Ž. McGaw L. J. (2022). Best Practice in the chemical characterisation of extracts used in pharmacological and toxicological research-The ConPhyMP-Guidelines. Front. Pharmacol. 13:953205. 10.3389/fphar.2022.953205 36176427 PMC9514875

[B15] Huedo-Medina T. B. Sánchez-Meca J. Marín-Martínez F. Botella J. (2006). Assessing heterogeneity in meta-analysis: Q statistic or I2 index? Psychol. Methods. 11(2):193–206. 10.1037/1082-989x.11.2.193 16784338

[B16] Langton C. R. Harmon Q. E. Baird D. D. (2024). Family history and uterine fibroid development in black and african American women. JAMA Netw. Open. 7(4):e244185. Conflict of Interest Disclosures: None reported. Epub 20240401. 10.1001/jamanetworkopen.2024.4185 38568693 PMC10993075

[B17] Laughlin-Tommaso S. K. Stewart E. A. (2018). Moving toward individualized medicine for uterine leiomyomas. Obstet. Gynecol. 132(4):961–971. 10.1097/aog.0000000000002785 30130343 PMC6153058

[B18] Lee S. Stewart E. A. (2023). New treatment options for nonsurgical management of uterine fibroids. Curr. Opin. Obstet. Gynecol. 35(4):288–293. 10.1097/gco.0000000000000880 37144584 PMC10330353

[B19] Liu Y. (2020). Effect of Shugan Sanjie Decoction on lesion volume and hemodynamics in patients with uterine fibroids. Pract. Clin. J. Integr. Traditional Chin. West. Med. 20 (13), 75–76. 10.13638/j.issn.1671-4040.2020.13.037

[B20] Ma W. (2023). Clinical effect of Shugan Sanjie Decoction combined with leuprelin on patients with uterine fibroids of Qi stagnation and blood stasis. Shenzhen J. Integr. Traditional Chin. West. Med. 33 (9), 42–45. 10.16458/j.cnki.1007-0893.2023.09.012

[B21] Manyonda I. Belli A. M. Lumsden M. A. Moss J. McKinnon W. Middleton L. J. (2020). Uterine-artery embolization or myomectomy for uterine fibroids. N. Engl. J. Med. 383(5):440–451. 10.1056/NEJMoa1914735 32726530

[B22] Marsh E. E. Wegienka G. Williams D. R. (2024). Uterine fibroids. Jama. 331(17):1492–1493. 10.1001/jama.2024.0447 38598205

[B23] Meng W. Lin W. L. Yeung W. F. Zhang Y. Ng E. H. Y. Lee Y. P. E. (2022). Randomized double-blind trial comparing low dose and conventional dose of a modified traditional herbal formula Guizhi Fuling Wan in women with symptomatic uterine fibroids. J. Ethnopharmacol. 283:114676. Epub 20210922. 10.1016/j.jep.2021.114676 34562564

[B24] Mitro S. D. Wise L. A. Waetjen L. E. Lee C. Zaritsky E. Harlow S. D. (2024). Hypertension, cardiovascular risk factors, and uterine fibroid Diagnosis in midlife. JAMA Netw. Open. 7(4):e246832. 10.1001/jamanetworkopen.2024.6832 38625699 PMC11022113

[B25] Ning G. Zhang X. Zhang Q. Wang Z. Liao H. (2020). Real-time and multimodality image-guided intelligent HIFU therapy for uterine fibroid. Theranostics. 10(10):4676–4693. Competing Interests: The authors have declared that no competing interest exists. Epub 20200326. 10.7150/thno.42830 32292522 PMC7150484

[B26] Page M. J. McKenzie J. E. Bossuyt P. M. Boutron I. Hoffmann T. C. Mulrow C. D. (2021). The PRISMA 2020 statement: an updated guideline for reporting systematic reviews. Bmj. 372:n71. Epub 2021/03/31. 10.1136/bmj.n71 33782057 PMC8005924

[B27] Shi S. Luo L. Peng F. Yu C. (2023). Potential mechanism of Taohong Siwu Decoction in uterine fibroid treatment based on integrated strategy of network pharmacology and experimental verification. Chin. Med. 18(1):95. The authors declare that they no competing interests. Epub 20230802. 10.1186/s13020-023-00809-6 37533095 PMC10398959

[B28] Silberzweig J. E. Powell D. K. Matsumoto A. H. Spies J. B. (2016). Management of uterine fibroids: a focus on uterine-sparing interventional techniques. Radiology. 280(3):675–692. 10.1148/radiol.2016141693 27533290

[B29] Sterne J. A. C. Savović J. Page M. J. Elbers R. G. Blencowe N. S. Boutron I. (2019). RoB 2: a revised tool for assessing risk of bias in randomised trials. 366:l4898, Available online at: www.icmje.org/coi_disclosure.pdfanddeclare . 10.1136/bmj.l4898 31462531

[B30] Stewart E. A. Cookson C. L. Gandolfo R. A. Schulze-Rath R. (2017). Epidemiology of uterine fibroids: a systematic review. Bjog. 124(10):1501–1512. Epub 20170513. 10.1111/1471-0528.14640 28296146

[B31] Stewart E. A. Laughlin-Tommaso S. K. Catherino W. H. Lalitkumar S. Gupta D. Vollenhoven B. (2016). Uterine fibroids. Nat. Rev. Dis. Prim. 2:16043. Epub 20160623. 10.1038/nrdp.2016.43 27335259

[B32] Stewart E. A. Missmer S. A. Rocca W. A. (2021). Moving beyond reflexive and prophylactic gynecologic surgery. Mayo Clin. Proc. 96(2):291–294. 10.1016/j.mayocp.2020.05.012 33549251 PMC8088594

[B33] Stewart E. A. Nowak R. A. (2022). Uterine fibroids: hiding in plain sight, 37, 16, 27. 10.1152/physiol.00013.2021 PMC8742728 34964688

[B34] Syed Y. Y. (2022). Relugolix/estradiol/norethisterone (norethindrone) acetate: a review in symptomatic uterine fibroids. Drugs. 82(15):1549–1556. 10.1007/s40265-022-01790-4 36331779 PMC9684252

[B35] Tulandi T. (2007). Treatment of uterine fibroids--is surgery obsolete? N. Engl. J. Med. 356(4):411–413. 10.1056/NEJMe068281 17251539

[B36] Vannuccini S. Petraglia F. Carmona F. Calaf J. Chapron C. (2024). The modern management of uterine fibroids-related abnormal uterine bleeding. Fertil. Steril. 122(1):20–30. 10.1016/j.fertnstert.2024.04.041 38723935

[B37] Walker C. L. Stewart E. A. (2005). Uterine fibroids: the elephant in the room. Science. 308(5728):1589–1592. 10.1126/science.1112063 15947177

[B38] Wang B. (2021). Effect of Shugan Sanjie decoction on TCM syndrome score and P level in patients with hysteromyoma Qi stagnation and blood-stagnation syndrome. Contemp. Med. 27 (11), 79–81.

[B39] Wu H. (2021). Therapeutic effect of Shugan Sanjie Decoction on the syndrome of uterine fibroid qi stagnation and blood stasis. Women's Health (30).

[B40] Wu Y. Zhang C. Chen Y. Luo Y. J. (2018). Association between acute mountain sickness (AMS) and age: a meta-analysis. Mil. Med. Res. 5(1):14. Ethics approval and consent to participate: not applicable. competing interests: The authors declare that they have no competing interests. Epub 20180511. 10.1186/s40779-018-0161-x 29747689 PMC5946480

[B41] Xu M. (2022). Clinical effect analysis of Shugan Sanjie Decoction in the treatment of uterine fibroids with Qi stagnation and blood stasis syndrome. Electron. J. Pract. Gynecol. Endocrinol. 9 (11), 72–74.

[B42] Yang Q. Ciebiera M. Bariani M. V. Ali M. Elkafas H. Boyer T. G. (2022). Comprehensive review of uterine fibroids: developmental origin, pathogenesis, and treatment. Endocr. Rev. 43(4):678–719. 10.1210/endrev/bnab039 34741454 PMC9277653

[B43] Zhai Y. (2017). Clinical study on the treatment of hysteromyoma with qi stagnation and blood stasis syndrome by shugan Sanjie tang. ACTA Chin. Med. 32(12):2516–2518. 10.16368/j.issn.1674-8999.2017.12.653

[B44] Zhang B. (2020). Clinical study on shugan Sanjie decoction in treating hysteromyoma with qi zhi xue yu zheng. Clin. study Shugan Sanjie Decoction Treat. hysteromyoma Qi Zhi Xue Yu Zheng 36 (23), 105–106.

[B45] Zhang F. (2023). Clinical observation of Shugan Sanjie Decoction treating syndrome of qi stagnation and blood stasis in uterine fibroids. Clin. observation Shugan Sanjie Decoction Treat. syndrome qi stagnation blood stasis uterine fibroids 39 (10), 1940–1941.

[B46] Zhang Y. Peng W. Clarke J. Zhishun L. (2010). Acupuncture for uterine fibroids. Cochrane Database Syst. Rev. 2010. 10.1002/14651858.CD007221.pub2 PMC11270531 20091625

[B47] Zhou L. (2019). Clinical observation on 50 cases of uterine fibroid qi stagnation and blood stasis syndrome treated by integrated traditional Chinese and Western medicine. Chin. J. Ethnomedicine Ethnopharmacy 28 (5), 86–88.

